# eRegistries: governance for electronic maternal and child health registries

**DOI:** 10.1186/s12884-016-1063-0

**Published:** 2016-09-23

**Authors:** Sonja L. Myhre, Jane Kaye, Lee A. Bygrave, Margunn Aanestad, Buthaina Ghanem, Patricia Mechael, J. Frederik Frøen

**Affiliations:** 1 Department of International Public Health, Norwegian Institute of Public Health, P.O. Box 4404, Nydalen, N-0403 Oslo Norway; 2 Centre for Health, Law and Emerging Technologies, Nuffield Department of Population Health, University of Oxford, Rosemary Rue Building, Old Road Campus, Headington, Oxford, OX3 7LF UK; 3 Department of Private Law, Faculty of Law, University of Oslo, Postboks 6706, St Olavs plass, 0130 Oslo, Norway; 4 Department of Informatics, University of Oslo, Gaustadalléen 23 B, N-0373 Oslo, Norway; 5 Palestinian National Institute of Public Health, Qaddoura Street, Ministry of Health Building, 1st Floor, Postbox 54812, Ramallah, Palestine; 6 School of Advanced International Studies, Johns Hopkins University, 1717 Massachusetts Ave, NW, Washington, DC 20036 USA; 7 HealthEnabled, Unit D11, Westlake Square, Westlake Drive, Westlake, Cape Town, South Africa 7945; 8 Centre for Intervention Science in Maternal and Child Health, University of Bergen, Postbox 78000, 5020 Bergen, Norway

**Keywords:** Ethics, Law, Data privacy, Security, Governance, Registry, Maternal and child health

## Abstract

**Background:**

The limited availability of maternal and child health data has limited progress in reducing mortality and morbidity among pregnant women and children. Global health agencies, leaders, and funders are prioritizing strategies that focus on acquiring high quality health data. Electronic maternal and child health registries (eRegistries) offer a systematic data collection and management approach that can serve as an entry point for preventive, curative and promotive health services. Due to the highly sensitive nature of reproductive health information, careful consideration must be accorded to privacy, access, and data security. In the third paper of the eRegistries Series, we report on the current landscape of ethical and legal governance for maternal and child health registries in developing countries.

**Methods:**

This research utilizes findings from two web-based surveys, completed in 2015 that targeted public health officials and health care providers in 76 countries with high global maternal and child mortality burden. A sample of 298 public health officials from 64 countries and 490 health care providers from 59 countries completed the online survey. Based on formative research in the development of the eRegistries Governance Guidance Toolkit, the surveys were designed to investigate topics related to maternal and child health registries including ethical and legal issues.

**Results:**

According to survey respondents, the prevailing legal landscape is characterized by inadequate data security safeguards and weak support for core privacy principles. Respondents from the majority of countries indicated that health information from medical records is typically protected by legislation although legislation dealing specifically or comprehensively with data privacy may not be in place. Health care provider trust in the privacy of health data at their own facilities is associated with the presence of security safeguards.

**Conclusion:**

Addressing legal requirements and ensuring that privacy and data security of women’s and children’s health information is protected is an ethical responsibility that must not be ignored or postponed, particularly where the need is greatest. Not only are the potential harm and unintended consequences of inaction serious for individuals, but they could impact public trust in health registries leading to decreased participation and compromised data integrity.

## Background

At the 2014 Maternal and Child Health Summit, World Bank Group President Jim Yong Kim proclaimed, “Our vision is to register every single pregnancy and every single birth by 2030” [1]. As the Millennium Development Goal (MDG) era draws to a close and the Sustainable Development Goals (SDG) are ushered in, a shift towards long-term investments, sustainable strategies, and infrastructure development have emerged as new priorities [2, 3]. Growing support for strengthening civil registration and vital statistics [4–6] and the call for more and better maternal health data in 2010 by leadership in eight global health agencies [7] all point to the need to improve data collection strategies in low and middle income countries (LMIC). Against this backdrop, in June 2015 the World Health Organization (WHO), the United States Agency for International Development (USAID), and the World Bank released *The Roadmap for Measurement and Accountability* and Post-2015 *5-Point Call to Action* that highlight strategies for improving data collection, analysis, access, and use [8]. The dearth of timely and accurate maternal and child health data has limited countries’ ability to measure progress in reducing maternal and child deaths worldwide but has galvanized leaders [9–11] and funders [12, 13] to prioritize strategies to acquire high quality maternal and child health data.

Electronic health registries (eRegistries) for maternal and child programs provide a unique approach given their potential to support both clinical and public health decision-making, enhance health care coverage, and improve health outcomes by providing individual data along the continuum of care that can pinpoint when, where, and why women encounter health problems [14–16]. Field studies and research applying the registry concept to maternal health have demonstrated promise [17, 18] in contrast with ad hoc, resource-intense surveys and statistical estimates of maternal mortality (i.e., MMR) that have been criticized for their inability to accurately assess MDG progress [19]. The burgeoning focus on measurement, monitoring and infrastructure and universal health coverage and equity are consistent with registry methodology that involves ongoing, population-based data collection that strengthens data availability, quality and use [16, 20].

While electronic maternal and child health registries compile comprehensive individual health data [14, 15, 21] Frost et al, personal communication, 2016, the highly sensitive nature of reproductive health information and the vulnerability of women and children living in LMICs demand careful consideration of privacy, confidentiality, and data security. The pace of the data revolution has outstripped the ability of existing laws and traditional approaches to address concerns introduced by digital technology [22–25]. Electronic health data, for example, are susceptible to intentional or inadvertent breaches of security with serious implications for individuals’ privacy that did not exist in the ‘paper era.’ The transition from paper to electronic records, thus, demands contemporary strategies that ensure patient privacy, confidentiality, and data security [26].

The *Principles for Digital Development*, an initiative involving WHO, the World Bank, USAID, the Bill and Melinda Gates Foundation, and a host of other international agencies, provide guidance on how to integrate best practices in ICT projects and specifically highlights the need to address privacy and security in their eighth principle [27]. Responsible data stewardship practices among communities [28] and ethical checklists for use in humanitarian operations [29] are two recent examples of guidance tools. However, despite the proliferation of health registries around the world, few publications provide an overarching framework or discuss approaches to ethical or governance issues specifically for registries [30–32]. To address this gap, the eRegistry Governance Guidance Toolkit (Frame 1) was developed to provide an overview of the ethical and legal issues pertaining to electronic registries and identify best practices that protect women and children’s health data in LMICs [33].

The research literature on ethical and governance issues in LMICs suggests that these countries face additional challenges compared to developed countries and may need to address different ethical and legal issues concerning electronic health registries due to a lack of capacity, training, and ICT expertise, along with low literacy rates, limited infrastructure and weak governance [34–36]. The WHO’s Global Observatory for eHealth series, for example, notes that LMICs face unique challenges in monitoring and managing eHealth data [37, 38] while a TrustLaw report on mHealth data privacy and security issues underscores the importance of culture and context [39]. A lack of clear policies, governance, and legislation has also been observed by researchers in LMIC countries [40, 41]. Ideally, local capacity is needed in public health, medical informatics, law, medical ethics, and privacy protection to address privacy and security issues.

Physical infrastructure limitations such as lack of rooms, partitions, or curtains may also negatively influence patient privacy in the context of health service provision. Resource-constrained settings may negatively impact attitudes and perspectives regarding medical confidentiality practices, particularly with respect to illiterate or poor populations [42, 43]. The concept of confidentiality in a medical context, for example, may also be understood and practiced differently depending on the setting due to differing cultural and social expectations regarding privacy [44].

The consequences of neglecting to address privacy or security issues that pertain to a maternal and child health registry in LMICs have the potential to compromise public trust. As a ‘public health good,’ a registry relies on trust, which is achieved and maintained by appropriate measures to protect individual privacy. Reproductive health data demand the highest level of care given that it may contain information on HIV status, pregnancy terminations, or other highly stigmatizing information [45]. Privacy breaches for health registries are particularly concerning given the sensitivity of this type of personal health data. Theft or internal disclosure, for example, may result in personal information being divulged for profit, intelligence, defamation, or embarrassment resulting in stigma, discrimination, exclusion, or persecution [46]. Privacy protections are regarded as a basic human right that can only be abrogated in cases where there is ample justification [47, 48].

Privacy protections need to consider both internal (e.g., negligent or malicious actions by health care providers), and external threats (e.g. hackers) to ensure that personal health data are only used for the intended purpose and accessed or disclosed to authorized personnel under strict controls. For example, a judgment issued by the European Court of Human Rights, (I v Finland, 2009), concluded that Finland had violated the European Convention on Human Rights, Article 8 given that hospital authorities had failed to adequately implement technological measures to ensure confidentiality of a patient’s medical data [49]. A report prepared by the International Telecommunications Union on cybersecurity in LMICs also emphasized the importance of security policies that are customized, continually optimized, and adapted to the stakeholders and the local environment in which they are implemented [50]. “Privacy by design” is one strategy that proactively incorporates security measures throughout the design of software or information systems via technological means such as access controls, passwords, and encryption [51].

Governance mechanisms also assume an important role [52]. Borrowing from the biobank literature, governance is defined by formal oversight mechanisms (i.e., regulatory bodies, legal instruments) and informal mechanisms (i.e., advisory boards, policies, guidance, professional values, and culture) that together guide decision-making, compliance, and policy development [53]. Governance may be developed to address a range of issues including accountability, transparency, redress, purpose specification, data collection limitations, secondary use of data,, security breach notifications, and data quality and integrity [46].

To assess the current perceptions and status of legal, privacy, and data security issues, public health officials and health care providers residing in 76 countries were invited to complete an online survey. Seventy-five of these countries, according to the Commission on Information and Accountability for Women’s and Children’s Health (CoIA), shoulder the greatest burden of maternal and child mortality [54] while the occupied Palestinian territory was included given the challenges related to healthcare access and political instability [55].

## Methods

This paper is based on findings from two web-based surveys that targeted public health officials and health care providers (i.e., midwives, nurses and doctors in reproductive, maternal, and child health). Based on formative research conducted in the development of the eRegistries Governance Guidance Toolkit (Frame 1), the aim of the surveys was to assess the current status of legal, privacy and security issues relevant to maternal and child health registries in LMICs.

### Frame 1: The eRegistries Governance Guidance Toolkit

The eRegistries Governance Guidance Toolkit [56] was developed to advise countries on how to proceed with the establishment, operation, use, and maintenance of an eRegistry for maternal and child health that is lawful and compliant with existing legal requirements, protective of women’s rights and privacy, and supportive of the public health aims of the registry. Formative research undertaken in the development of this toolkit involved an extensive review of standards, methods, and procedures established by health registry systems (i.e., cancer, chronic disease, diabetes, and clinical) and vital statistics (i.e., birth registration). The Toolkit was reviewed by experts in registry law, informatics, and public health.

The Toolkit identifies best practices, discusses benefits of legislation, regulations, and guidelines, and provides guidance for countries that can be adapted to local contexts. The Toolkit outlines the essential governance components including: purpose specification, legal, fiscal, and operational responsibility, reporting requirements and enforceability, data quality, data security, confidentiality policies, and data access, and public engagement. The Toolkit considers relevant international instruments, conventions, and declarations that focus on human rights, privacy, data protection, and data security as these may provide useful information, particularly for LMICs that lack national legislation or enforcement bodies.

eRegistries for maternal and child health must function within the legal framework where they operate which can involve legal requirements pertaining to medical research, public health, women’s and children’s rights, and information law (i.e., data protection law, ethical use of data). One challenge of developing governance guidance in a global context is the inherent diversity in how countries approach law, ethics, and health. Social and cultural differences in how privacy, confidentiality, and security are managed may influence laws, policies and protocols. The Toolkit encourages country level adaptation and advises against transplanting legal language or documents from one country to another. Instead, country level policies should be rooted in their own institutional fabric. Translated adaptations, for example, often fail to embrace subtle social or cultural mores that may affect acceptance.

### Survey methods

The survey recruitment strategy consisted of individualized email invitations to reproductive, maternal, newborn and child health (RMNCH) medical and health organizations, Ministries of Health, Institutes of Public Health, and other related government offices (e.g., statistics bureaus, RMNCH departments, etc.) working in any of the 75 countries identified as the highest burden countries by CoIA and the occupied Palestinian territory, collectively called CoIA countries in this paper. Surveys and invitations were available in English, French and Portuguese. (The surveys are available upon request from the first author.)

The public health official survey sample consists of 298 individuals from 64 countries (84 % of the invited countries). A total of 470 health care providers from 59 countries (78 % of invited countries) participated in the health care provider survey. Among public health officials, approximately two-thirds worked at the national or regional level in a Ministry or public health institute or agency. Among health care providers, the professional breakdown included 170 (37 %) doctors, 66 14 %) nurses, 149 (32 %) midwives and 81 (17 %) other RMNCH professionals. Eighty percent (*n* = 341) of the health care providers worked in urban or suburban areas while one- fifth (88) were in rural or isolated areas. Among the health care providers, 198 (44 %) reported working at a public or private hospital, 46 (10 %) worked at a district facility, community health post or maternity home, 91 (20 %) were employed at a public health organization, 62 (14 %) were employed at a MoH, and 49 (11 %) selected ‘other.’

A breakdown of the public health official survey respondents by the six WHO regions found that 37 of 42 CoIA countries (88 %) were represented from the African region (88 %), 4 out of 6 (67 %) in the America region, 2 out of 5 (40 %) CoIA countries in the European region, 5 out of 6 (83 %) CoIA countries in the South-East Asian region, 6 out of 7 countries (86 %) in the Western Pacific region, and all ten CoIA countries in the Eastern Mediterranean region (100 %). Among health care provider survey respondents in CoIA countries, 32 out of 42 (76 %) countries were represented in the African region, 8 out of 10 (80 %) countries in the Eastern Mediterranean region, 2 out of 5 (40 %) European countries, 4 out of 6 (67 %) of the South-East Asian countries, and all countries in the America (6/6) and Western Pacific region (7/7) were represented. The personalized invitations contained live links to the online surveys and requested that individuals participate and share the survey with peers, colleagues and professional networks (i.e., a snowball sampling recruitment method) in order to boost the sample size via a referral strategy. Paper-based surveys were made available in some circumstances. Launched in November 2013, responses were accepted until February 2015. Repeated efforts were attempted for all non-responsive countries.

Thematic areas measured by the survey included national registry infrastructure, legal and ethical issues, data security, health care service provision, reporting and dissemination practices, data quality, and data usage. This paper focuses on the ethical and legal domains while results concerning the other topics are reported elsewhere [14, 15, 57] Frost et al, personal communication, 2016. The public health official and health care provider surveys contained overlap of core thematic content but also included questions adapted specifically to the different target groups in order to capture their unique professional and workplace perspectives. The public health official survey, for example, included detailed questions on civil registration systems and data utilization whereas the health care provider survey contained specific items on service provision and data reporting from a health care provider perspective.

### Ethical review

The survey was reviewed by the Regional Committees for Medical and Health Research Ethics in Norway and received a letter of exemption given that all information collected was fully anonymous (Reference number: IRB 0000 1870). All respondents were informed that their answers were completely anonymous and that they could withdraw from the survey at any time.

### Data analysis

Descriptive statistics were used to present most findings while generalized linear models (PROC GLM) were used to assess more complex associations. Exact confidence intervals were generated from tables. All analyses were done using SAS 9.4. Responses from the public health officials were collapsed to the country level while health care providers were analyzed on the individual level. This strategy was specifically chosen to avoid masking the inherent variability among health care provider settings while facilitating national level assessments with public health official responses.

With regard to data security measures, the surveys asked about physical, technical, and administrative safeguards for protecting electronic registry medical records. Due to missing data among health care provider responses, only public health official data are reported. With regard to the question on level of trust that health care providers have in their own facility’s security, respondents were asked to rate how comfortable they would be having their own data stored at their work facility using a five-item Likert scale ranging from very comfortable to very uncomfortable.

## Results

This paper describes the perceptions and perspectives of health care providers and public health officials from 76 LMICs with respect to privacy, access, and data security of personal health information.

### Current legal privacy protections

Human rights generally and the right to privacy specifically are usually enshrined in legislation or regulations that are passed by legislative bodies and can be readily enforced. Survey respondents were asked if their country had laws or regulations that protect a person’s privacy or confidentiality concerning their personal health data (i.e., information or medical records). Public health officials from 69 % of the 61 responding countries (*n* = 42; 95 % CI: 56–80) reported that their country had legislation protecting individual privacy.

### Access

The survey explored different forms of access ranging from individual-level access by women to access among health care professionals or others directly or indirectly involved in a patient’s care. In the majority of countries (*n* = 48; 79 %; 95 % CI: 66–88), public health officials indicated that individuals have the right to access their own medical records.

**Fig. 1:**
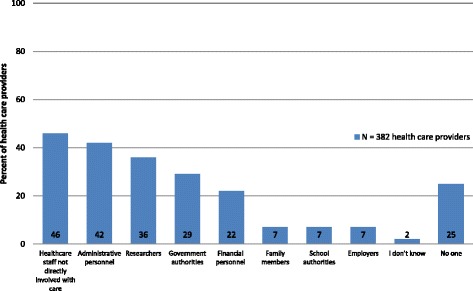
Access to patient health records without patient consent. Legend: Percent of responding health care providers indicating access to patient health care records without consent, by category

Access by others was assessed by asking respondents, *“Aside from health professionals directly involved in a patient’s care, who else has access to patient medical records without patient consent?”* Health care providers indicated that many actors within and outside the health system have access to medical records without patient consent. The survey results found that health care providers working in diverse settings indicated that access to data by actors not directly involved with the patient included: other health care providers not directly involved with the patient’s care (*n* = 174; 45 %; 95 % CI: 40–51), administrative staff (*n* = 163; 43 %; 95 % CI: 38–48), financial staff (*n* = 82; 21 %; 95 % CI: 17–26), government (*n* = 111; 29 %: 95 % CI: 25–34), school (*n* = 25; 7 %; 95 % CI: 4–10), employers (*n* = 28; 7 %; 95 % CI: 5–10), researchers (*n* = 137; 36 %: 95 % CI: 21–41), and family members (*n* = 26; 7 %; 95 % CI: 4–10) (Fig. 1). In circumstances in which patients are asked to provide consent to share health information, health care providers mentioned multiple methods such as written (*n* = 256; 67 %; 95 % CI: 62–72), verbal (*n* = 160; 42 %; 95 % CI: 37–47) and biometric approval (*n* = 46; 12 %; 95 % CI: 9–16).

Respondents were asked about the secondary use of registry data for research purposes. Ninety-four percent of countries (*n* = 46; 95 % CI: 83–99), according to public health officials, indicated that researchers could request access to the data whereas only 63 % (*n* = 31; 95 % CI: 18–45) reported that internal health personnel could obtain access. In 61 % (*n* = 30) of countries (95 % CI: 46–75), the general public could apply to use national health data for research purposes.

### Data security

**Fig. 2:**
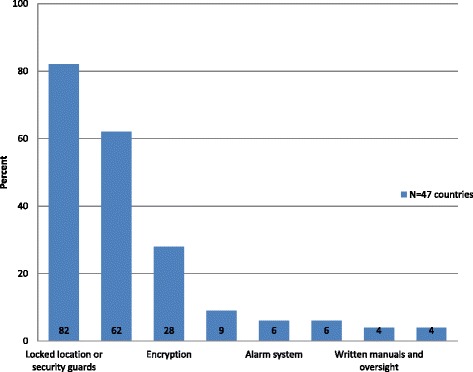
Data security safeguards. Legend: Percent of countries, based on public health official survey

Security measures designed to protect health information are categorized as physical, technical, or administrative safeguards. Data security questions in the public health official survey were only answered by individuals indicating that they worked in data management by using a skip logic question before this section (*n* = 47). According to their responses, most countries still rely on traditional physical safeguards typically used for paper systems such as locked buildings and security guards (Fig. 2). Alarm systems were reported by very few countries (*n* = 3; 6 %; 95 % CI: 1–18)). Nine percent of countries (*n* = 4; 95 % CI: 2–20) have no physical safeguards at all. The use of passwords to access data and files was the most commonly reported technical safeguard noted by public health officials in 62 % of the countries (*n* = 29; 95 % CI: 46–75). Encryption - a method of protecting data in transit that converts data into another form that can only be understood by authorized parties - was reported in use by 27 % of countries (*n* = 13; 95 % CI: 16–43). Restricted access, considered an administrative security measure, was reported in 89 % of countries (*n* = 42; 95 % CI: 79–98). Very few countries reported the use of written security manuals or monitoring committees.

Another strategy to ensure data security is to store health data separately from unique identifier codes or numbers. According to public health officials, among countries that stored individual level data (*n* = 37), approximately half (*n* = 18; 49 %; 95 % CI: 32–66)) indicated that data and codes were stored together, 35 % stored data and codes separately (*n* = 13; 95 % CI: 20–53), and 16 % reported that they did not know how data was stored (*n* = 6; 95 % CI: 6–32).

Among health care providers, 63 % (*n* = 236; 95 % CI: 58–68) indicated that they were comfortable with the privacy and security of storing their own health records at the facility in which they worked, one-quarter reported feeling very comfortable and 20 % (*n* = 73; 95 % CI: 16–24) reported feeling very or slightly uncomfortable. Upon closer examination, individual comfort level was associated with the presence of security safeguards such as locked buildings or security guards (*p* < .0001) and password protection (*p* < .0028). Of note, Rwanda stood out as an exemplary country given that all 20 respondents selected the highest level of comfort with regard to storing their personal health information at their work facility.

## Discussion

### Privacy

The majority of countries have national constitutions that address individual privacy [38] and, similarly, medical professional codes of ethics, international instruments (i.e., the Helsinki Declaration) and the Hippocratic oath embrace patient confidentiality. In addition, a regulation impact assessment of ten African countries documented that many legal frameworks recognize and protect an individual’s right to privacy [58]. Data privacy law, for example, may fall under both civil codes and telecommunication legislation.

Although the survey questions did not specify data privacy legislation, a comparison of the respondents answering that their country had general privacy legislation indicates that a much smaller fraction have actually passed comprehensive data privacy legislation, i.e., legislation that specifically regulates various stages of the processing of personal data with the principal object of safeguarding privacy (57). Data privacy legislation has been passed in more than 100 countries worldwide based on numerous sources including books [59], reports [58, 60], published articles [61, 62], online web pages [63] and international law directories [64–66], but further analysis reveals that only 21 % of these countries are among the 75 high burden countries identified by CoIA countries. In other words, of the 106 countries that have successfully passed data privacy laws, 22 are among those with the highest burden. Pending data privacy legislation is currently under consideration in ten additional high burden countries signifying a growing trend [58, 61].

In addition to the importance of the adoption of data privacy laws in high burden countries, data privacy is a process that involves data protection regimes and enforcement bodies to regulate compliance [67]. Although neither survey addressed this issue, it is worth noting that there is pressure from Europe to introduce data privacy regimes that meet the adequacy standards set by the European Data Protection Directive 95/46/EC. Thus, data privacy law is part of a larger process requiring attention to enforcement by data protection authorities. Given that this survey only assessed one aspect of data privacy, a more thorough evaluation is needed of the impact of data privacy legislation on public health officials’ and health care providers’ experiences.

### Access

The high proportion of the 76 high burden countries reporting individual access to their own personal health data suggests that many of these countries have legally enshrined their value of patients’ rights and autonomy. Findings concerning access to patient records, however, indicate that a substantial number of actors outside of the health system have access to medical records without patient consent. In particular, access to patient medical records by government authorities, for example, may have significant privacy implications for women. Access to registry data by government authorities, law enforcement, and or the judicial system may threaten women’s privacy and hinder registry participation for fear of self-incrimination as discussed in Frame 2 on Brazil’s pregnancy registration law. The potential violation of confidentiality, considered the core of the physician-patient privilege, may prompt women to avoid formal health systems in favor of less regulated options.

### Frame 2: Brazil’s pregnancy registration law

The Brazilian Ministry of Health’s enactment of a health law establishing a national pregnancy registration system raises important lessons regarding issues that can seriously impact public trust. The presumed impetus for Brazil’s registry law was the 2011 case, *Alyne v Brazil* that was brought before the UN Committee on the Elimination of Discrimination against Women Committee by the Center for Reproductive Health Rights and Advocacia Cidadã pelos Direitos Humanos on behalf of the Brazilian woman’s family that died during childbirth due to allegedly inadequate maternal health care [68]. Ruling in favor of the deceased’s family, Brazil was found in violation of international obligations to provide adequate access to maternal health care and urged to take steps to remedy their system.

On December 26, 2011, Brazil’s president passed an emergency Provisional Measure 557, the ‘National System for Registration, Surveillance and Monitoring of Pregnant and Postpartum Women for the Prevention of Maternal Mortality.’ The timing sidestepped congressional approval suggesting anticipated opposition. The stated aim of the statute was to improve access, coverage, and quality of maternal health care in order to reduce Brazil’s high number of maternal deaths.

The main point of contention is the obligatory nature of participation combined with the potential for self- incrimination if a pregnant woman elects to terminate her pregnancy [69]. Brazil’s restrictive abortion law only allows abortions when the mother’s life is in danger, the pregnancy is the result of rape, or severe genetic abnormalities are detected. Consequently, a woman is subject to prosecution if she terminates her pregnancy. The discord between Brazil’s abortion law with mandatory universal pregnancy registration poses obvious challenges given that the legal parameters of the pregnancy registry include compulsory participation without informed consent or opt-out options [70]. While it may be argued that health registries legitimately perform best with universal participation and implied consent, the obligatory nature of Brazil’s system is not counterbalanced by legal provisions that protect a woman from incrimination or ensure optimal health care.

One solution is to restrict the use of registry data aside from the intended purpose for public health. This can be achieved by clearly stating the registry purpose in a legal mandate with parameters that prevent personal health information from being used to incriminate participants in a court of law. Despite the proclaimed intention of MP 557 to reduce maternal deaths, the unintended consequence may be an increase in maternal deaths due to avoidance of early prenatal care or an increase in unsafe abortion procedures. The structure of MP 557 ultimately erodes the essential trust between women and health care providers. Consequently, women may choose to not seek medical care in order to avoid being registered. Thus, not protecting women’s privacy undermines public trust and may result in reduced public support for health registries.

### Security

Security is defined as strategies such as safeguards, policies, or protocols through which access or sharing of patient health information by stakeholders is controlled and protected from intentional or unintentional disclosure to unauthorized persons, and from loss, destruction or alternation [40]. Security controls applied to electronic data can take many forms including anonymity techniques, encryption, authentication systems, access control models, access policies, user roles, audit logs, and education and training of employees [71].

As reported by public health officials, the physical, administrative, and technical data security safeguards currently in use do not appear to adequately safeguard women’s and children’s highly sensitive health information. A common assumption is that since electronic information systems are in a nascent stage in many LMICs [72], the skills to access systems unlawfully are similarly underdeveloped. Yet, this discounts potential threats from outside a country [73]. Such general skepticism of potential threats may reflect an overall lack of concern and subsequent inaction by many e- and mHealth projects in LMICs. Moreover, data and information security is maintained differently in resource-constrained countries given the limited ICT capacity, training, and resources. As a result, privacy and security issues have not received the same attention in countries with emerging electronic health systems. In addition, a workforce inexperienced or untrained in safe data practices may not fully appreciate the far-reaching implications of security breaches.

Another rationale for not prioritizing security issues is the notion that health needs outweigh privacy concerns in LMIC countries [46]. Moreover, there may be a presumption that it is too premature to address these issues prior to security legislation or regulation being adopted. The potential for harm and unintended consequences of ignoring legal and ethical issues, however, is considerable on both an individual and societal level. Compromising the privacy of an individual’s sensitive health information can have devastating consequences for the individual and his/her family and on a larger scale, could undermine trust in electronic health information systems in general thereby undercutting efforts to improve health.

### Implications for practice and future research

Initiating eRegistries for women and children into countries with the greatest need necessitates due diligence to ensure that the ethical and legal considerations are attended to in order to protect women and children’s health data. The current gaps in protections for privacy and data security suggest that internal governance should be crafted to address these issues. Future research should continue to investigate the influence of culture, literacy rates, privacy, infrastructure and capacity in LMICs [36]. A notable challenge of developing guidance in a global context is the inherent diversity in how countries approach law, ethics, and health. Cultural beliefs and religious practices may significantly influence approaches to confidentiality, privacy, and security [67].

A country’s legal, ethical, and cultural parameters will also affect the processes, priorities, and policies that are developed as noted in the Palestine experience in Frame 3 [74]. Thus, it is essential to carefully evaluate and assess the legal, regulatory, ethical, social, and cultural environment and adapt guidance accordingly. Transplanting legal language or documentation from one country to another can be problematic. Country-level policies should be rooted in their own social and institutional fabric as translated adaptations are typically not able to embrace subtle social and cultural mores that can negatively impact acceptance and compliance.

### Frame 3: Mapping Palestine’s legal landscape for an MCH eRegistry

Presently, Palestine is in the process of establishing and implementing an eRegistry for maternal and child health in the absence of formal legislation or presidential decree. Due to the unresolved and unpredictable political situation and historically overlapping legal traditions, navigating the Palestinian legal system is complicated. The Palestinian Basic Law (passed in 2002 and amended in 2003 and 2005) functions as a temporary constitution while the Palestinian Legislative Council (i.e., Parliament) is the legislative branch with limited ability to act or govern.

Mapping the legal, regulatory, and ethical landscape using a global situation analysis tool tailored for the Palestinian context was the first step taken to identify gaps and actions necessary to ensure an ethical and lawful framework for an eRegistry for maternal and child health. The mapping exercise revealed that Palestine has limited legislation relevant to health registries. Palestine’s civil registration law enacted in 1966 and amended in 2001, according to a UN technical report, for example, is relevant to health registries.

Palestine does not have a specific data privacy law although provisions in the Penal Law No. 16 of 1960 indicate that disclosing confidential information is unlawful and can result in imprisonment for up to three years. As well, there is mention of honoring data confidentiality and individuals’ privacy in Article 4 of the General Statistics Law (2000) [66]. Although there are no health registry laws in place, the Public Health Law (2004) does address general maternal and child health issues in Articles 4 and 5 [75].

Ensuring that data security, data protection and women’s privacy are fully protected in the eRegistry poses challenges given this legal environment but also provides opportunities to recommend comprehensive governance structures accompanied by robust national protocols and guidelines. Technical solutions embedded in the eRegistry platform, like the ‘privacy by design’ framework developed in Canada [51] ensure privacy through de-identification strategies as well as regulate access through authorization protocols, encrypt health information to assure anonymity, and address insider threats to data privacy via auditing strategies. Local and customary patient-provider practices and relationships as well as social norms must also be considered in order to develop culturally competent approaches.

Conceptually, these efforts have recently been described in terms of data stewardship that contribute to a ‘chain of trust’ [76] that can facilitate good will and public trust. Depicting this process as a successive set of steps reinforces the importance of maintaining communication with stakeholders regarding the responsibilities of data stewardship of women’s health information.

### Strengths and limitations

There are advantages and challenges inherent to web-based surveys. Advantages include timeliness, cost, and viability of obtaining responses from a global target group. Web-based recruitment strategies facilitated achieving a large number of responses from a diverse set of countries which is one strength of the study. Variability in the number of responses from each country, however, limits generalizability. Given that survey participation relied on internet access, some individuals may not have participated due to poor or unavailable internet connectivity thus limiting representativeness. Survey responses with a high proportion of missing values were not included in the analysis. Finally, external validation of survey items was challenging due to the evolving state of data privacy policies and regulations in LMICs. Despite these limitations, the study explores compelling issues that merit further inquiry.

## Conclusion

Reflecting on the essential elements for health registries, one researcher commented that the “confidentiality and ethical issues can often decide the success of the registry [77].” Privacy and security woven into health registry systems must bolster public trust, promote adoption, and maintain individual confidentiality. Data should not be used in a way that compromises a patient’s rights to confidentiality and privacy. Given the value, opportunity, and potential of maternal and child health registry data to contribute to improved maternal and child health, it is imperative to address privacy by building in core principles and protocols combined with oversight and accountability mechanisms [51]. This research hopes to shed light on the challenge of balancing individual privacy without deterring responsible data use. The field must invest in better defining and understanding risk while at the same time not losing sight of the public good and practical potential of maximizing health data analysis. The transition from the MDG to the SDGs, learning from early experiences in implementing eRegistries, and a maturing approach to protecting privacy and security in a digital age, provides a unique opportunity for both vision and responsible engagement. Underlying efforts to leverage innovation and new technology, as in all other movements to improve health, is the responsibility to respect universal human rights.

## Abbreviations

CoIA: Commission on Information and Accountability for Women’s and Children’s Health
ICT: Information and communication technology
LMIC: Low and middle income country
MCH: Maternal and child health
MDG: Millennium Development Goal
MMR: Maternal mortality ratio
RMNCH: Reproductive, maternal, newborn, and child health
SDG: Sustainable Development Goal
UN: United Nations
USAID: United States Agency for International Development
WHO: World Health Organization
